# Promote or Inhibit: Economic Goal Pressure and Residents' Health

**DOI:** 10.3389/fpubh.2021.725957

**Published:** 2021-07-26

**Authors:** Min Zhong, Peng Wang, Ming Ji, Xi-Hao Zeng, Hong-Xiang Wei

**Affiliations:** ^1^College of Economics, Jinan University, Guangzhou, China; ^2^College of Economics and Management, Nanning Normal University, Nanning, China

**Keywords:** economic goal pressure, residents' health, environmental pollution, mechanism, heterogeneity

## Abstract

This paper aimed to identify the relationship between the pressure to reach economic growth targets and residents' health by applying a panel fixed effects model, a Sobel-Goodman mediation effects test and a regulatory effects model to the inland provinces of China. The empirical results verify that the pressure to reach economic growth targets in these regions reduces the level of residents' health. Moreover, the effect in developing regions is significantly stronger than that in developed regions, and the effect in the northern region is significantly stronger than that in the southern region. The mediation effects test found that the pressure to reach economic growth targets has led to an upsurge in PM2.5 concentration and an increase in the output of industrial solid waste, thereby threatening residents' health. The regulatory effects model highlights that enhancing public awareness could weaken the negative impact of the pressure to reach economic growth targets on residents' health, while the expansion of industrial production will aggravate the negative impact. In the process of economic growth, the government should set reasonable economic growth targets, pay attention to the construction of the environmental protection legal system, implement energy- conservation and emission reduction measures and increase public awareness of environmental protection to ensure residents' health.

## Introduction

The main purpose of this paper is to explore whether the economic growth target (measured by the difference between the GDP estimate and the target value of the government work report) set at the beginning of the year in various administrative regions in China affect residents' health (the death rate and moderate to severe health conditions for children under 5 years of age). If there is an effect, is it promoting or reducing resident' health? What is the mechanism of the effect? Unlike the estimated economic growth target of the United States, the economic growth target of China typically has highly restrictive characteristics. Because of vertical intergovernmental relations, the target value set in the actual operation of the lower-level government is still too high after the Chinese central government has set the standard for growth. Meanwhile, the performance appraisal of the Chinese government is mainly based on economic growth in past periods, which may lead local governments to attain economic growth at the expense of the local environment and energy requirements. In fact, since the 40th anniversary of the reform and opening up, China has indeed achieved and sustained high-speed economic growth, which has caused increasingly serious environmental pollution and led to residents' health problems. Relevant data show that China has 213,000 pulmonary heart disease patients and 1.5 million chronic bronchitis patients each year due to environmental pollution. The number of deaths is second in the world (1), which causes the direct health effect losses equivalent to 10% of the GDP. Residents' health is the foundation for sustained and stable economic and social development. Similarly, economic and social development affect residents' health. It is vital to transform the method of assessing economic growth so that residents' health and behavior is taken into account by the government. The optimization of the performance evaluation system is the most direct way to guide the behavior of local governments, and setting the target of GDP growth can partly reflect this guidance. This study is beneficial for decision makers who need to pay attention to the impact of the pressure to reach economic growth targets on residents' health. The government should establish a multi-indicator and decentralized assessment system to ensure residents' health, pay attention to the non-economic value of the environment to the region, weaken the prominence of the GDP target, increase environmental governance and improve environmental governance capabilities.

For developing countries and less developed countries (LDCs), the balance between economic growth and residents' health is even more difficult to keep. As other developing countries and LDCs may not have strong hierarchical systems of government due to differences in government systems, there is no pressure to set economic growth targets, and the trend of developing countries seeking economic growth will not essentially change. However, because of technological constraints in developing countries and LDCs, economic growth can only rely on the advantages of their natural resource. The massive consumption of non-renewable energy, backward environmental governance, inadequate medical and health standards, and the deterioration of residents' health have hindered the sustained and stable development of the economy. And fluctuations in energy prices will increase the uncertainty of domestic economic policies (2). For example, according to the 2016 Global Burden of Diseases, Injuries, and Risk Factors Research Report released by The Lancet in 2018, there is a large gap of the medical and health levels (HAQ index) between developing countries and developed countries. The top 20 countries in terms of medical capacity are mainly concentrated in Europe, and low- and middle-level countries need to pay more to improve the quality of medical care. In addition, developed countries have also gone through the journey of “pollution first” on economic growth and residents' health. For example, the “Fog Capital” in the United Kingdom and the “Great Lakes” area in the United States have fundamentally solved the vicious dilemma of economic development and residents' health.

In the past years, with the Chinese economy growing rapidly and stably, the negative correlation between economic growth pressure and residents' health, as well as the concurrent effects of environmental pollution, etc., have all been shown intuitively and empirically. Whether the pressure to reach economic growth targets has a statistical impact on residents' health, how much impact it has, and the transmission mechanism needs to be verified by a scientific system. Can mild (moderate) pressure to reach economic growth targets take economic growth and residents' health into account? At different levels of economic development, does the pressure to reach economic growth targets have different effects on residents' health? For different economic development models, is there any difference in the influence and transmission mechanism of the pressure set by the economic growth target? In response to the above problems, the marginal contribution of this paper is as follows. First, the empirical results show that the pressure to reach economic growth targets in Chinese provinces and cities has a significant negative effect on residents' health, and it is more pronounced in less developed regions and northern regions. The reason may be that these regions are mostly resource-based cities. Second, the mediation effect test verified the intermediate relationship between air and solid waste pollution, and air pollution has a greater impact on residents' health. In addition, the regulatory effect test verified that the negative effects of local residents' environmental awareness and industrial production activities on economic growth pressure have been alleviated. Therefore, regional governments can initiate residents' environmental protection education and industrial structure transformation and to upgrade to a more balanced relationship between economic growth and residents' health.

This study uses data from 30 inland provinces and cities in China from 2003 to 2019 as a sample to explore the direct effects of regional the pressure to reach economic growth targets on residents' health and the mediating effects of environmental pollution. The organization of this article is as follows. Section Literature Review reviews the existing literature, section Economy Growth and Residents' Health Model builds the theoretical model of this paper based on the Solow model, section Methodology describes the measurement model of this paper, section Data introduces indicator data, section Empirical Results analyses the empirical results, and the seventh section is the conclusion.

## Literature Review

Economic activities will lead to changes in the general environment of lives and residents' lifestyles, which will lead to health problems (3–5). The existing literature on the relationship between the economy and residents' health mainly focuses on economic growth. Few studies have focused on the pressure to reach economic growth targets, and the research conclusions mainly hold the viewpoint that economic growth has a negative effect on the health of local residents. Ebenstein et al. (6) used Chinese city-level data to investigate the relationship between economic growth and life expectancy, and the study showed that there is indeed a positive correlation between the two. Bombardini and Li (7) used China as an example to explore the relationship between the Chinese municipal economy and trade opening and infant mortality. The study found that infant mortality caused by respiratory diseases was positively correlated with the output value of local high-polluting export companies. Pierce and Schott (8) took the United States as an example to study the relationship between the normal trading partnership (PNTR) and the US mortality rate. The results show that economic trade will significantly increase the suicide rate in the United States. However, at the same time, some economic activities such as the construction of public infrastructure may also promote residents' health by effectively reducing transportation costs, optimizing the allocation of medical resources (9), and improving the efficiency of medical system assistance (10). Bell (11) took India as an example and found that rural roads can improve the health of villagers through the impact assessment of rural road projects in India. Similarly, Banerjee and Sachdeva (12) used India's large-scale national road construction data to show that large-scale road construction can increase the consumption of health care by women and families by reducing travel costs.

Economic growth is an eternal topic in economics, but the environmental problems brought about by rapid economic growth have greatly reduced the positive effects of income growth due to the reduction of residents' health. At the same time, government intervention, such as the improvement of system quality (13), can effectively coordinate the relationship between the economy and the environment. In the early and mid-industrialization periods, economic growth was accompanied by a continuous increase in the proportion of industry and manufacturing. Limited by production technology, it was bound to bring tremendous pressure on the environment. The Environmental Kuznets Curve (EKC) is the most classic theory for the study of economic growth and environmental pollution. This theory shows that with the increase in per capita income, environmental pollution mainly presents an inverted U-shaped change that first rises and then falls (14). Buehn and Farzanegan (15) fitted the CO_2_, SO_2_, and N_2_O emissions of 122 countries with per capita GDP and verified the EKC hypothesis. Alam et al. (16) studied the relationship between Chinese CO_2_ emissions and income, and found that it conforms to the EKC hypothesis. Luo et al. (17) studied the relationship between CO_2_ emissions and economic growth in G20 countries, and found that developing countries are still mainly in the increasing stage at the left end of the inverted U-shaped curve, while developed countries are mainly in the inverted U-shaped curve at the decreasing right end. At the same time, some scholars have reached different conclusions from the EKC through data fitting. Auffhammer and Carson (18) used Chinese provincial data to study the relationship between CO_2_ emissions and economic growth and found that the relationship between the two is N-like. Musolesi et al. (19) distinguished between high-income and low-income countries and found after comparative analysis that the CO_2_ emissions of high-income countries and GDP per capita showed an N-shaped change, while the CO_2_ emissions of low- and middle-income countries and GDP per capita showed a linear increasing trend.

Existing studies consistently show that environmental pollution, especially air pollution, is negatively correlated with residents' health. For developed countries, Pedersen et al. (20) took Europe as an example. The study found that there is a significant positive correlation between air pollution in Europe and low birth-weight infants and that the impact of PM10 on low birth-weight is higher than that of PM2.5. Currie and Neidell (21) used micro-individual data from California in the 1990s and found that PM10 and CO concentrations are significantly correlated with infant mortality. In the same way, research on developing countries has also reached similar conclusions. Chen et al. (22) took 90 cities located in the north and south of the Huaihe River in China from 1981 to 2000 as examples. They studied the relationship between the city's daily total suspended particulate matter concentration and the death rate of residents. The resulting increase in the concentration of suspended particulate matter may reduce the average life expectancy of 500 million residents in northern China by 5 years. Tanaka (23) took the Chinese 1998 “Acid Rain Control Zone and Sulfur Dioxide Pollution Control Zone Division Plan” policy as the node, and found that improving air pollution can effectively reduce infant mortality. Arceo et al. (24) took Mexico as an example. Studies have shown that air pollution can cause infant deaths due to respiratory and cardiovascular diseases, and infant mortality has a significant non-linear relationship with CO concentration.

Above all, although economic growth (especially the pressure of economic growth) may result in environmental pollution and lead to a decline in residents' health, as economic activities involve a wide range of activities, there may be other causes that impact residents' health, such as the construction of public facilities, which has a positive effect on residents' health. Therefore, the total effect of the pressure to reach economic growth targets on residents' health remains to be explored, and the mediating role of environmental pollution remains to be verified. Therefore, this article will focus on the above two tasks.

## Economy Growth and Residents' Health Model

Health is regarded as an important part of human capital, and human capital is one of the important elements of economic growth (25). To explore the impact of the pressure to reach economic growth targets on residents' health, this paper introduces the human capital production function proposed by Bloom on the basis of the Solow model and decomposes human capital into healthy human capital and other human capital (26, 27), The production function can be preliminarily expressed as:

(1) $$\begin{array}{r} Y=K^{\alpha }L^{\beta }e^{\gamma_{1}s+\gamma_{2}h} \end{array}$$

Where Y is the output level, *K* is the capital factor input, *L* is the labor factor input, *h* is the healthy human capital level, s is the level of human capital other than health, and α, β, and γ represent capital, labor and manpower, respectively, the parameters of the capital element. These are the impact of human capital on output is exponential, indicating that the marginal wage income (β) obtained by labor is a function of human capital (including healthy human capital and other human capital).

Environmental pollution caused by economic activities will affect the final output by affecting the physical health of the labor force; that is, environmental pollution will bring externalities. On this basis, this article adds a description of the environment to formula (1). The function of pollution externality *X*(·) is modified to:

(2) $$\begin{array}{r} Y=K^{\alpha }L^{\beta }X\left(\cdot \right)e^{\gamma_{1}s+\gamma_{2}h} \end{array}$$

In order to facilitate the calculation, the logarithmization of formula (2) can be obtained:

(3) $$\begin{array}{r} \ln\;Y=\alpha \text{lnK+}\beta \text{lnL+ln}\left(X\left(\cdot \right)\right)+\gamma_{1}s+\gamma_{2}h \end{array}$$

Environmental pollution mainly depends on three factors, namely, natural degradation, government governance (G) and corporate pollution discharge (Z), as shown in formula (4). Natural degradation is determined by the basic characteristics of pollutants and is regarded as completely exogenous. It is assumed that the environmental pollution stock *X* is degraded at an average annual rate of η. Government governance depends on the government's governance capabilities and the government's material (funds, expertise) support. The greater the pressure of the government's economic growth is, the more the government's material endowment will be tilted toward economic activities, to a certain extent, thereby reducing the effect of government governance ω, However, if the government's environmental governance capacity is stronger, the negative effects brought by the government's environmental governance resources can be alleviated to a certain extent. Corporate pollution emissions are mainly affected by its industry and government environmental supervision. The greater the pressure on the government's economic growth is, the higher the degree of acquiescence to corporate polluting behavior will be. Heutel (28) and Annicchiarico and Dio (29) assume that the company's pollution emissions are a certain percentage of output μ; the greater the pressure on economic growth is, the higher μ, as shown in formula (5).

(4) $$\begin{array}{r} X_{t}=\frac{X_{t-1}^{\eta }Z_{t}}{G_{t}^{\omega }} \end{array}$$

(5) $$\begin{array}{r} Z=\mu Y \end{array}$$

By converting formula (4) into a static formula, and after performing logarithmic processing with formulas (5–7) can be obtained:

(6) $$\begin{array}{r} \left(1-\eta \right)\ln\;X=\ln\;Z-\omega \ln\;G \end{array}$$

(7) $$\begin{array}{r} \ln\;Z=\ln\;\mu +\ln\;Y \end{array}$$

Combining formulas (3), (6), and (7), after replacing *lnY* and *lnZ*, the healthy human capital *h* can be solved as:

(8) $$\begin{array}{r} h=\frac{1}{\gamma_{2}}\left[-\ln\;X^{\eta }\left(\cdot \right)+\omega \ln\;G-\ln\;\mu -\alpha \ln\;K-\beta \ln\;L-\gamma_{1}s\right] \end{array}$$

To explore the direct impact of the government's economic goal setting pressure on residents' health, let the healthy human capital *h* have a partial derivative of the government governance *G* and corporate pollution emission ratio, and formulas (9) and (10) can be obtained, respectively. According to formula (9), the greater the pressure on the government's economic goals, the less resources will be invested in the environmental field. *G* declines, and *h* changes in the same direction as *G*, which is not conducive to residents' health; for the same reason, according to formula (10), the greater the pressure on the government's economic goals, the more likely the government is to acquiesce to the company's polluting behavior to ensure economic growth μ rises, and μ changes in the opposite direction as *h*, which will harm residents' health. In summary, when the pressure to reach economic growth targets is too great, the government will ensure that the annual economic target reaches the expected value and meet the relevant political assessment standards, which will be detrimental to the health of the residents. Of course, this model also has certain limitations. Some regions may also undergo industrial transformation and upgrading due to the pressure set by economic growth targets. The new momentum of economic growth after the transformation can ensure residents' health while ensuring economic growth, and because of this article, no indicators to measure regional industrial transformation and upgrading are added, so such effects cannot be assessed.

(9) $$\begin{array}{r} \frac{\partial h}{\partial G}=\frac{1}{\gamma_{2}}\cdot \frac{\omega }{G↓}\;>0 \end{array}$$

(10) $$\begin{array}{r} \frac{\partial h}{\partial \mu }=\frac{1}{\gamma_{2}}\left(-\frac{1}{\mu ↑}\right)\;<0 \end{array}$$

On this basis, the following research hypotheses are proposed:

The pressure of the government's economic goal setting will have a negative impact on residents' health because of the government's behavioral preferences.

To explore the role of environmental pollution in the government's economic goal setting pressure on residents' health, we combine formulas (6) and (7) to replace *lnZ*, and formula (11) can be obtained, which is the logarithm of the environmental pollution stock *X* Taking the partial derivative of the logarithm *lnY* of the output level, we can obtain formula (12). According to formula (12), the degree of environmental pollution changes in the same direction as output growth. The faster the output growth rate, the more serious the environmental pollution.

(11) $$\begin{array}{r} \ln\;X=\frac{1}{1-\eta }\cdot \left(\ln\;Y-\omega \ln\;G+\ln\;\mu \right) \end{array}$$

(12) $$\begin{array}{r} \frac{\partial \ln\;X}{\partial \ln\;Y}=\frac{1}{1-\eta }\;>0 \end{array}$$

On the other hand, let the healthy human capital h in formula (8) obtain the partial derivative of the environmental pollution stock *X*, and then formula (13) can be obtained. From formula (13), it can be seen that environmental pollution and residents' health change in the opposite direction, and environmental pollution will directly lead to a decline of residents' health.

(13) $$\begin{array}{r} \frac{\partial h}{\partial X}=-\frac{\eta }{X}\;<0 \end{array}$$

On this basis, the following research hypothesis is proposed:

The pressure set by the government's economic goals results in environmental pollution, which has a negative impact on residents' health.

## Methodology

### Panel Fixed Effects Model

Based on the above theoretical model argumentation, this paper constructs a panel fixed effects model to test the relationship between the pressure to reach economic growth targets and residents' health as well as influencing factors. With reference to the existing related research, the panel fixed effects model set in this paper is as follows:

(14) $$\begin{array}{r} {Health}_{it}=\alpha_{0}+\alpha_{1}Pressure_{it}+\alpha_{2}X_{it}+\mu_{i}+\varepsilon_{it} \end{array}$$

In model (14), pressure represents the pressure brought by the setting of economic growth goals; Health represents the health status of residents; *X*_*it*_*i* is the control variable; α_1_ and α_2_ represent the coefficients of the core explanatory variable and the control variable, respectively; μ_*i*_ represents samples from different provinces Individual fixed effect; ε_*it*_ is a random error term; *i* and *t* represent province and year, respectively.

### Sobel-Goodman Mediation Tests

In order to further explore the relationship between the pressure to reach economic growth targets and residents' health and its mechanism of action, this paper uses a hierarchical regression method to establish an intermediary mechanism test model as follows:

(15) $$\begin{array}{r} {Health}_{it}=\alpha_{0}+\alpha_{1}Pressure_{it}+\alpha_{2}X_{it}+\mu_{i}+\varepsilon_{it} \end{array}$$

(16) $$\begin{array}{r} M_{it}=\beta_{0}+\beta_{1}pressure_{it}+\beta_{2}X_{it}+\mu_{i}+\varepsilon_{it} \end{array}$$

(17) $$\begin{array}{r} Health_{it}=\gamma_{0}+\gamma_{1}Pressure_{it}+\gamma_{2}M_{it}+\gamma_{3}X_{it}+\mu_{i}+\varepsilon_{it} \end{array}$$

(18) $$\begin{array}{r} Health_{it}=\theta_{0}+\theta_{1}Pressure_{it}^{*}R_{it}+\theta_{2}X_{it}+\mu_{i}+\varepsilon_{it} \end{array}$$

In the intermediary effect model, *Pressure*_*it*_ represents the pressure brought by the setting of economic growth goals; Health represents residents' health; *X*_*it*_ is a control variable; *M*_*it*_ represents the intermediary variable that represents the living environment of residents, including the PM2.5 concentration in the air *Pwair*_*it*_ and industry solid waste discharge *Solid*_*it*_;*R*_*it*_ represents the regulatory variable, *ln*_*paware* and *ln*_*induspro* reflecting environment awareness and industrial production; $Pressure_{it}^{*}R_{it}$ is interactive item. α_1_ and α_2_, respectively, represent the core explanatory variable and the coefficient of the control variable of formula (15), respectively; β_1_ and β_2_ represent the coefficient of the core explanatory variable and the control variable of formula (16), respectively; γ_1_ and γ_2_ represent the formula (17) core explanatory variables and coefficients of the control variables, respectively; θ_1_ and θ_2_ represent the coefficients of the core explanatory variables and control variables of formula (18), respectively; μ_*i*_ represents the individual fixed effects of samples in different provinces; ε_*it*_ is the random error term; *i* and *t* represent the province and year, respectively.

## Data

This paper selects annual data from 2003 to 2019 for all inland provinces, autonomous regions, and four municipalities in China except Tibet, with a total of 510 observations. Due to the lack of some environmental data before 2003, to ensure the validity of the research, the time node of the empirical research is set to start in 2003. The data in this article comes from the “China Statistical Yearbook,” “China Environmental Statistics Yearbook,” and reports on the work of the National Bureau of Statistics and provincial governments. This paper uses the trend function interpolation method to calculate the GDP growth forecast for the next year as the expected economic growth rate using the actual GDP growth rate for each consecutive 5 years, and then subtracts the economic growth target and the expected economic growth rate to obtain the economic. The growth target pressure index *Pressure*_*it*_ serves as the core explanatory variable. The malnutrition rate of children under five is used to measure residents' health. This is a negative indicator. The larger the indicator is, the lower residents' health is. A large number of empirical studies have shown that economic growth has a positive effect on residents' health (30). However, the impact of economic growth on the environment shows an inverted U-shaped trend (31), which represents damage to the environment in the early stage of industrialization; Furthermore, when reaching the end of industrialization, technological innovation reduces the damage to the environment by production less and less. The pressure to reach economic growth targets will affect the industrial structure and inhibit green technological innovation (32), thereby causing environmental pollution. Therefore, this article focuses on the pressure to reach economic growth targets, and studies whether this pressure promotes or inhibits residents' health.

Since the relationship between the pressure to reach economic growth targets and residents' health may be affected by external factors, this paper selects six control variables to strengthen the credibility of the model. The first is the government's fiscal revenue (*Grec*), which reflects the government's ability to transfer payments. It directly affects the government's investment in public goods, infrastructure, and residents' health (33), as well as the degree of investment in the governance of the ecological environment. The second is fiscal expenditure (*Fe*), which reflects the government's fiscal decentralization, has a stimulating effect on economic growth targets (34), and is also an important factor in government investment in health (35). The pressure on economic growth targets may cause the government to relax environmental controls on industrial enterprises to achieve short-term goals. The third is education level (Edu), which is used to represent human capital. The improvement of education level means the improvement of regional technology level, which can significantly and efficiently promote economic growth (36, 37), which is an important reference element for setting economic growth goals; at the same time, education level and health are closely related (38). The fourth is labor wage (*Laborwage*), which determines the spending power of workers. The higher the salary is, the greater the propensity to invest in health is, and the more health level of residents will rise (39). The fifth is the total amount of imports and exports (*Export*), which measures the degree of opening up of a region and can effectively reflect the status of regional economic development and the degree of openness of society. The sixth is the policy variable (Policy), which is measured by the time point when the Chinese Environmental Protection Law was formally implemented the construction of dummy variables representing 2015. The promulgation of the Environmental Protection Law is accompanied by the advancement of a series of environmental protection policies, which makes the government must adhere to sustainable development (40) by coordinating the pressure to reach economic growth targets and residents' health. Due to the lack of individual data, this article uses interpolation to fill in missing data; when used for empirical purposes, part of the data is processed by logarithm.

**Figure 1:**
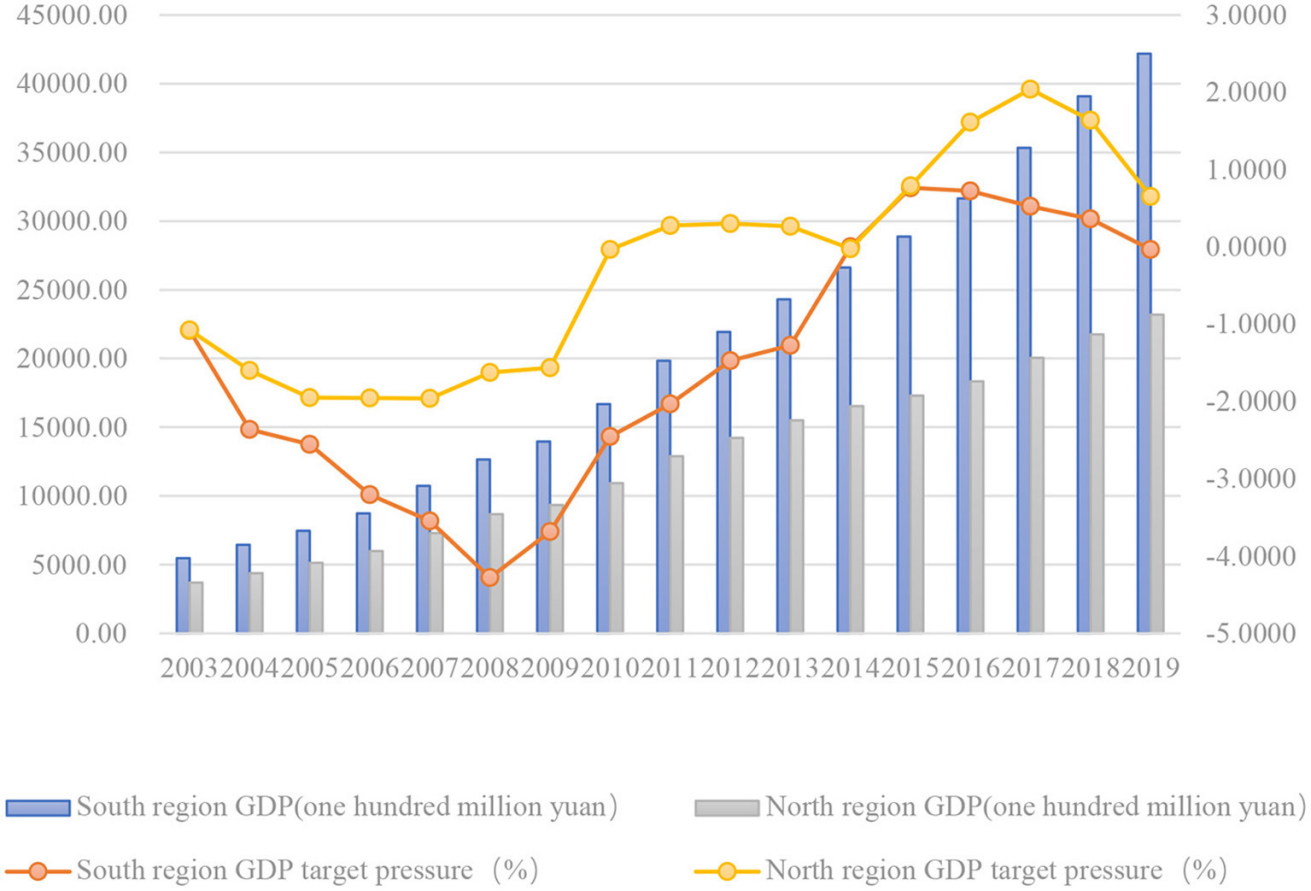
GDP and GDP target pressure in south region and north region.

**Figure 2:**
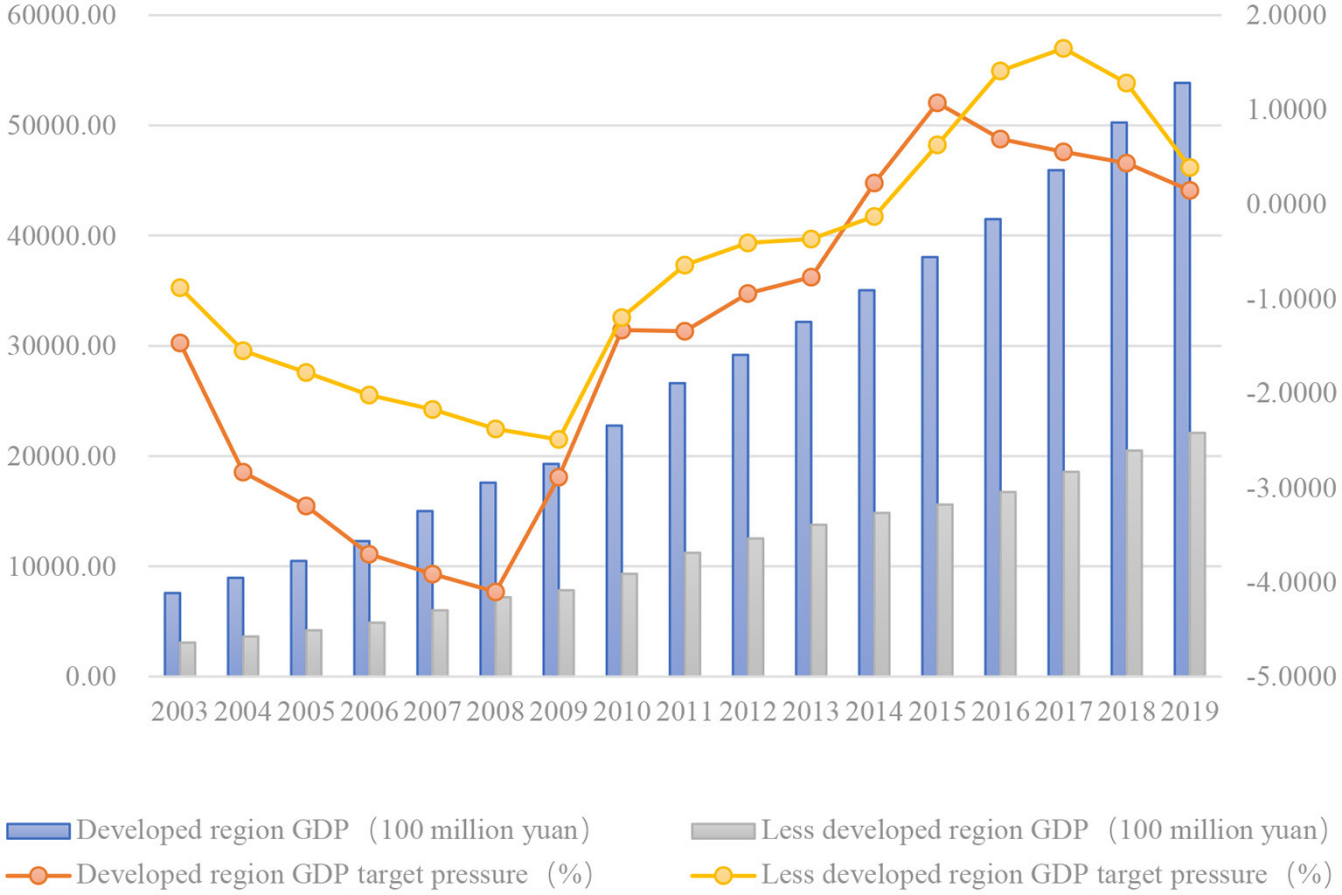
GDP and GDP target pressure in developed region and less developed region.

Figures 1, 2 reflects the change trend of the total GDP of the sample by region and the change of the pressure to reach economic growth targets. Judging from the overall trend reflected in the figure, the total GDP of each region has maintained a growth trend, and the target pressure of economic growth showed a downward trend before 2008. The period from 2008 to 2014 was the period of rising of the target pressure of economic growth, and until 2015, the pressure has eased in the future. Figure 1 shows the comparison of the data between the southern provinces and the northern provinces. The total GDP and growth rate of the southern provinces is higher as a whole, while the pressure to reach economic growth targets of the northern provinces is greater, and it continues to exist until ~2014. Figure 2 is a comparison of data between economically developed and less developed regions. The figure shows that the total GDP of developed regions is much higher than that of less developed regions, while less developed regions face greater pressure on economic growth targets, reaching a peak in 2017. The economic pressure in developed regions is relatively small.

**Figure 3:**
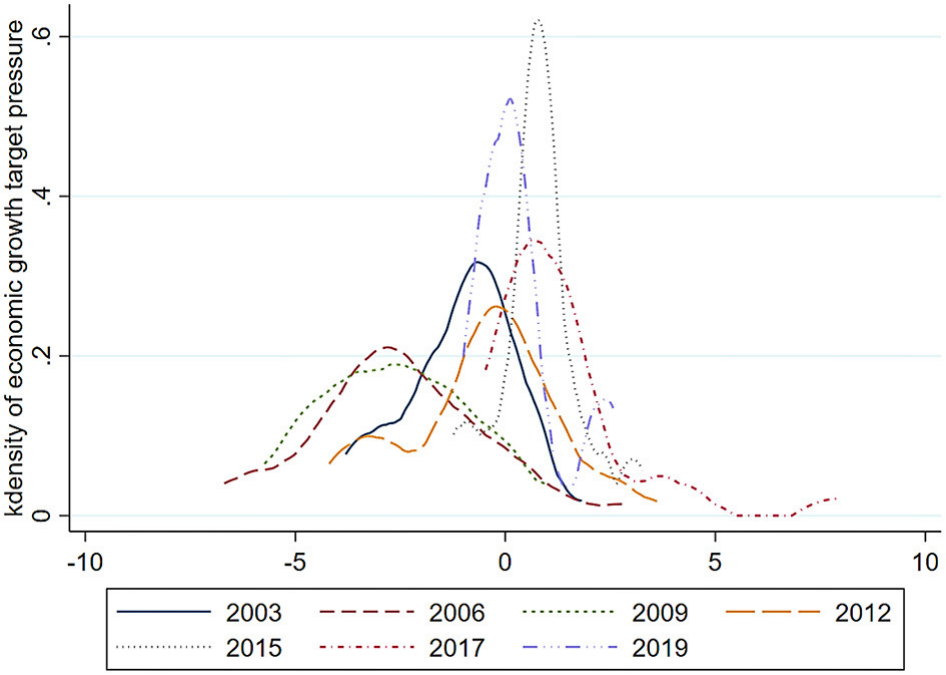
Kdensity of the pressure to reach economic growth targets.

Figure 3 shows the nuclear density map of the pressure to reach economic growth targets faced by various provinces in different years. In Figure 3, the nuclear density curve shows a rightward shifting trend, and the peak value shows a trend of first decreasing and then increasing over time. It is basically stable in 2015, and the abscissa was in the range of 0–2. This shows that in the early years, the pressure to reach economic growth targets was small or there were fewer provinces with the pressure to reach economic growth targets; however, after 2015, the pressure to reach economic growth targets was increasing, or most provinces had the pressure to reach economic growth targets.

Table 1 shows the descriptive statistics of the variables. The average value of residents' health indicators is 2.043, the minimum is 0.06, and the maximum is 16.66. This shows that the time and space of residents' health level is quite different, which may be related to health policy and environmental protection policy. Regarding the pressure to reach economic growth targets, the minimum and maximum values are −6.69 and 7.99, respectively, which are extremely different. The main reason is that different provinces and regions face different economic development conditions and face different levels of pressure. During the study period, the government's fiscal expenditures changed little and were basically stable; however, the government revenue was relatively large, which may be related to changes in taxation policies. The education level indicator tends to be stable, with small changes, while the labor compensation and total import and export changes are relatively large, which is also highly related to regional economic development.

**Table 1:** Descriptive statistics of the variables.

**Variables**	**Obs**	**Mean**	**Std. Dev**.	**Min**	**Max**
*Health*	510	2.043	2.025	0.060	16.660
*Pressure*	510	−0.877	2.177	−6.690	7.990
ln _*grec*	510	16.480	1.132	12.613	21.472
ln _*fe*	510	16.877	0.990	13.868	18.969
ln _*edu*	510	2.161	0.116	1.798	2.543
ln _*laberwag*	510	8.496	1.042	5.350	10.732
ln _*export*	510	17.148	1.707	12.734	20.811

## Empirical Results

Before conducting empirical analysis, it is necessary to test the stationarity of the data sample to avoid spurious regression in the model. Table 2 shows the ADF test on the important variables involved in the model, using the LLC test (41) and the IPS (42) test, which are two different methods to ensure the robustness of the results. The LLC test allows for different intercept terms and time trends, as well as heteroscedasticity and autocorrelation; IPS is more sensitive to limiting trend settings. The results show that there is no unit root in either the residents' health indicators or the pressure to reach economic growth targets indicators. Other important explanatory variables are also significant at the 5% confidence level. Therefore, the overall data tend to be stable, and regression analysis can be performed.

**Table 2:** Panel unit root tests.

**Variables**	**LLC unit – root test**		**IPS unit – root test**	
	**Adjusted t**	***p* − value**	**Z − t − tilde − bar**	***p* − value**
*Health*	−11.1104	0.0014	−8.8833	0.0000
*Pressure*	−14.0485	0.0000	−2.9364	0.0017
ln _*grec*	−16.6579	0.0000	−5.7398	0.0000
ln _*fe*	−6.2819	0.0000	−2.6140	0.0045
ln _*edu*	−4.4268	0.0258	−4.5849	0.0000
ln _*laberwag*	−8.3140	0.0001	−1.7761	0.0379
ln _*export*	−11.0336	0.0000	−5.2395	0.0000
*pwair*	−10.6798	0.0000	−5.6987	0.0000
ln _*solid*	−6.3954	0.0014	−2.2124	0.0135
ln _*paware*	−11.4883	0.0007	−4.3998	0.0000
ln _*induspro*	−13.3044	0.0000	−8.8325	0.0000

### Regression Analysis Results

The first column of Table 3 shows the results of the regression estimation of the pressure to reach economic growth targets on the residents' health level after controlling for the influence of other possible factors. There is an inverse relationship between the pressure to reach economic growth targets and the residents' health; the regression coefficient is 0.0939, which is significant at the 1% confidence level. This suggests that for every unit increase in the pressure to reach economic growth targets, the residents' health level drops by 0.0939 units. The pressure to reach economic growth targets stimulates the government to grasp economic growth, and the substitution effect of economic growth on residents' health is far greater than the income effect, and will generally reduce residents' health. In addition to the core explanatory variables, the level of government fiscal revenue and the degree of opening to the outside world have a significant positive impact on residents' health, with a significance level of 5%. The increase in fiscal revenue has improved the government's ability to transfer payments, stimulated investment in medical care, and affected residents' health. Expanding the degree of opening to the outside world can improve the regional industrial structure, promote technological innovation (43), curb pollution emissions, and improve health.

**Table 3:** Regression analysis and heterogeneity analysis results.

**Variables**	**Health**				
	**Total**	**Developed**	**Developing**	**North**	**South**
*Pressure*	0.0939***	0.0920	0.0931***	0.0960**	0.104*
	(0.0312)	(0.0574)	(0.0305)	(0.0385)	(0.0560)
ln _*grec*	−0.510**	−0.462*	−0.660	−0.686	−0.403
	(0.230)	(0.230)	(0.437)	(0.457)	(0.230)
ln _*fe*	−0.285	0.856	−0.334	−0.482	−0.0698
	(0.527)	(1.307)	(0.652)	(0.961)	(0.884)
ln _*edu*	0.397	−8.099	4.117**	2.199	−1.514
	(3.593)	(9.266)	(1.717)	(2.016)	(6.641)
*lnlaberwag*	−0.245	−0.972	−0.338	0.171	−0.475
	(0.635)	(1.551)	(0.931)	(1.041)	(0.949)
*lnexport*	−0.695**	−0.660*	−0.713	−0.869*	−0.614**
	(0.292)	(0.308)	(0.419)	(0.441)	(0.265)
*Policy*	0.182	0.299	0.134	0.0266	0.229
	(0.165)	(0.192)	(0.228)	(0.246)	(0.167)
*Constant*	28.42***	33.93**	23.86***	29.34***	28.38***
	(4.648)	(12.40)	(3.675)	(6.763)	(6.345)
*Observations*	509	170	339	254	255
*R*−*sq*	0.468	0.464	0.486	0.462	0.488
*Number*	30	10	20	15	15

Notes: ***, **, and * respectively indicates significance at the 1%, 5% and 10% level. Standard error in parentheses.

### Heterogeneity Analysis

The second to fifth columns of Table 3 analyze the samples according to the degree of economic development and geographic heterogeneity. As shown in Table 3, columns 2 and 3 reflect the regression results of the panel fixed effects model for developed and less developed regions. According to the GDP ranking of each province in the most recent year, the top ten are listed as developed regions, and the rest are unified and included in the statistics of less developed regions. The results show that the pressure to reach economic growth targets in developed regions does not have a significant effect on residents' health. There are two possible reasons. On the one hand, the pressure on economic growth targets in developed regions is relatively small, and there is even little pressure on economic growth targets in individual provinces. On the other hand, developed regions mainly rely on high-tech industries, high-quality service industries, the strong implementation of green development, and a relatively high emphasis on residents' health. However, for less developed regions, the negative effect of the pressure to reach economic growth targets on residents' health is significant at the 1% confidence level, with a coefficient of 0.0931. Less developed regions do not have a solid foundation for development as developed regions do. To achieve the economic growth targets set, the government may rely on industrial pull in the short term, ignoring environmental impacts on residents' health. In addition, although human capital variables have a significant effect, human capital in undeveloped areas is easily siphoned off by developed areas, resulting in insufficient economic growth potential and more pressure.

Columns 4 and 5 of Table 3 reflect the regression results of subsamples in southern and northern China, respectively. There are obvious differences in the economic development of Chinese regions; thus, the sample is divided into two parts according to the geographical dividing line of north and south China. The results show that the negative impact of the pressure to reach economic growth targets in the northern region on the health level is 0.096, with a significance level of 5%, while the southern region is also negatively affected, with an impact coefficient of 0.104 and a confidence level of 10%; that is, the northern region's impact is more obvious. The possible reasons are that, on the one hand, most of the southern regions of the country have a subtropical monsoon climate, with a better ecological environment, and most of the industries are light manufacturing industries, which are more suitable for residents' lives than in the north and can partially alleviate the pressure of economic growth targets and thus affect residents' health. On the other hand, more areas in the north are industrial or resource-mining areas with abundant mineral resources. In the early years, the resource-driven economic growth model harmed the environment, making it difficult to manage growth, and intensified the pressure on economic growth targets, which influences residents' health.

### Robustness Check

To ensure the reliability of the empirical results, this paper conducts a robustness test, mainly using different estimation methods and different sample ranges to compare with the basic results to examine whether the test results are consistent.

The generalized least squares method (GLS) is used to eliminate the autocorrelation of the random error term of the panel fixed-effects model, and then perform regression. According to the estimation results in columns one and two in Table 4, under the estimation of generalized least squares regression, the effect of the pressure to reach economic growth targets on residents' health is still negative, which is consistent with the estimated results of the panel fixed effects model, but the coefficients are slightly changed. This can show that the results of the estimation of the relationship between the pressure to reach economic growth targets and residents' health in this article are robust. In addition, considering the characteristics of different stages of Chinese economic development, economic development in the early years was mainly to promote growth; however, in recent years it has been emphasized that high-quality economic development, development speed and quality go hand in hand, which means that the theme of green development has been incorporated into the national strategy (44). This paper narrows the time frame of the study to 2003–2010 to test whether the above estimation results are robust. Column 3 and column 4 of Table 4 are the results of 30 provinces and cities in China from 2003 to 2010, respectively, using a panel fixed effects model and generalized least squares method. The results show that the pressure to reach economic growth targets still has a significant negative impact on residents' health. The influence coefficients are 0.0808 and 0.0669, respectively, which ensure the reliability and robustness of the empirical results in this paper.

**Table 4:** Robustness check.

	**Health**		**Health**	
	**GLS**		**Changing the Length of Time**	
*Pressure*	0.0421***	0.0213***	0.0808*	0.0669**
	(0.00462)	(0.00552)	(0.0416)	(0.0327)
ln _*grec*	−0.228***	−0.148***	−0.438	−0.108
	(0.0181)	(0.0156)	(0.299)	(0.111)
ln _*fe*	−0.599***	−0.668***	0.935	−0.705***
	(0.0411)	(0.0444)	(0.932)	(0.181)
ln _*edu*	−4.803***	−4.868***	4.980	−8.262***
	(0.213)	(0.251)	(4.599)	(0.803)
*lnlaberwag*		0.122***	−1.215	0.173
		(0.0459)	(1.399)	(0.147)
*lnexport*		−0.145***	−1.611***	−0.240***
		(0.0250)	(0.488)	(0.0576)
*Constant*	26.15***	27.46***	20.80**	35.76***
	(1.024)	(1.234)	(8.990)	(3.529)
*Observations*	510	510	240	240
*Number*	30	30	30	30

Notes: ***, **, and * respectively indicates significance at the 1%, 5% and 10% level. Standard error in parentheses.

### Mechanism Analysis

#### Mediation Effect

The previous theoretical analysis puts forward the hypothesis that the pressure set by the government's economic goals is transmitted through environmental pollution, which has a negative impact on residents' health. To verify this hypothesis, this article further uses the mediation effect model to test whether the mechanism proposed in the hypothesis exists and is effective. Refer to Table 5 for the estimated results of the mediation effect model. The first and second columns represent the regression analysis of the core explanatory variable pressure on the two selected mediation variables (air quality and industrial solid waste emissions), and the impact coefficients are found to be 0.0895 and −0.0115. However, they are not significant enough. A possible reason is that environmental indicators are also affected by other uncontrollable factors such as environmental protection propaganda, individual behavior habits and other variables. Further analysis of the third and fourth columns of Table 5 showed that the intermediary indicators were incorporated into the overall fixed effects model to construct formula (17). The results show that not only does the pressure to reach economic growth targets index has a significant negative impact on residents' health, the impact coefficients are 0.0913 and 0.0788, with a confidence level of 5%, but also air quality and industrial solid waste emissions have had a significant impact on residents' health. With reference to Wen's (45) intermediary mechanism test steps and determination methods, this article supplements the use of the Sobel test to further determine the existence of the intermediary effect. According to the results in Table 6, the *p*-values of the Sobel statistic are all <0.1, which indicates that the intermediary variables selected in this article are effective. Therefore, the effect of the intermediary mechanism can be further analyzed. According to the intermediary effect model, the product of the β_1_ and γ_2_ coefficients of the intermediary variables *Pwair* and *Ln*_*solid* in formulas (16) and (17) are all positive numbers, and the coefficients of γ_1_ are both significant, which indicates that the pressure to reach economic growth targets can produce some mediating effects by affecting the environmental quality, thereby affecting residents' health, and the mediating effects accounted for 2.74 and 16.1%, respectively. Specifically, the pressure to reach economic growth targets has caused some areas to pursue excessive economic growth and ignore the environment, which has led to an increase in PM2.5 concentration and an increase in solid waste emissions, which ultimately inhibits residents' health; thus, verified the previous hypothesis. Before entering the stage of high-quality development, China's early economic growth was mainly driven by industry, and there were still insufficient environmental governance. Secondly, there are GDP assessment tasks in various provinces and regions, and the governments of some provinces have a bad tendency to be GDP-only, which inhibits innovation to a certain extent, leading to the existence of a “priority development, later governance” model. Combined with the descriptive analysis of the target pressure on economic growth in this article, this model was very common in the early stage, and it formed a situation where the pressure on the early economic growth target was less. At the same time, air quality and industrial emissions cannot be effectively controlled, and the health of residents has also been negatively affected. However, after 2015, the 13th Five-Year Plan has elevated green development to a national strategic position. Provinces and cities have to change the model of first development and then governance, and coordinate economic growth with environmental governance. The pressure on economic growth targets is relatively obvious. Under the collaborative governance model, residents' health has been effectively improved.

**Table 5:** Mediation analysis.

**Variables**	**Pwair**	**Health**	**Ln_solid**	**Health**
*Pressure*	0.0895	0.0913***	−0.0115	0.0788**
	(0.199)	(0.0298)	(0.00880)	(0.0335)
*Pwair*		0.0287**		
		(0.0116)		
ln _*solid*				−1.315***
				(0.368)
ln _*grec*	1.750	−0.560**	0.120	−0.352**
	(1.135)	(0.247)	(0.0830)	(0.158)
ln _*fe*	3.510	−0.385	0.477**	0.343
	(2.258)	(0.497)	(0.203)	(0.575)
ln _*edu*	−18.29**	0.921	−0.133	0.222
	(8.761)	(3.391)	(0.547)	(3.280)
*lnlaberwag*	4.960	−0.387	0.0289	−0.207
	(3.020)	(0.638)	(0.259)	(0.585)
*lnexport*	−4.535***	−0.565*	−0.0182	−0.719***
	(1.635)	(0.329)	(0.107)	(0.233)
*Policy*	−13.33***	0.564**	−0.157***	−0.0243
	(1.271)	(0.267)	(0.0536)	(0.120)
*Constant*	38.39*	27.32***	−1.041	27.05***
	(19.19)	(4.283)	(1.583)	(4.081)
*Observations*	509	509	509	509
*R*−*sq*	0.392	0.479	0.679	0.529
*Number*	30	30	30	30

Notes: ***, **, and * respectively indicates significance at the 1%, 5% and 10% level. Standard error in parentheses.

**Table 6:** Sobel-Goodman mediation tests.

**Variables**	**Sobel**		**Goodman − 1**		**Goodman − 2**	
	***z*-value**	***p*-value**	***z*-value**	***p*-value**	***z*-value**	***p*-value**
ln _*solid*	−2.029	0.042	−1.992	0.046	−2.069	0.039
*pwair*	−1.731	0.084	−1.706	0.088	−1.756	0.079

#### Regulatory Effect

When studying the impact of government economic goal setting pressure on residents' health, this paper finds that some potential factors can have an effect on this impact. Therefore, this paper chooses residents' environmental awareness (ln _*paware*) and industrial production (ln _*induspro*) as the regulatory variables to test whether they can adjust the relationship between the pressure to reach economic growth targets and residents' health. The environmental awareness index is measured by dividing the area's annual domestic waste removal and transportation volume by the total population, reflecting the per capita waste production volume. The industrial production index is expressed by the total industrial output value of the year. The interaction terms between the regulatory variables and the core explanatory variables are introduced. According to the results in Table 7, the coefficient of the interaction term (pressure^*^ln_*paware*) after adding the control variables is significant at the 1% confidence level, with a coefficient of −0.0298, which indicates that the public's environmental awareness The increase can alleviate the inhibitory effect of the pressure to reach economic growth targets on residents' health to a certain extent; the coefficient of the interaction term (pressure^*^ln_*induspro*) is 0.013, which is also significant at the 1% confidence level, which means that the increase in industrial output value is certain. To a certain extent, it promotes the restraining ability of the pressure to reach economic growth targets on residents' health. As the country pays attention to environmental protection awareness, encourages conservation, reduces garbage, and promotes garbage classification, residents' awareness of environmental protection has increased significantly. This has significantly improved the quality of the residents' living environment. Secondly, the state's stricter control of industrial emissions has further promoted the improvement of the environment and restrained the trend of excessive industrial growth regardless of the environment. The enhancement of environmental protection awareness and industrial production management has enabled residents' health to be maintained at a good level under the pressure of existing economic targets.

**Table 7:** Regulatory effect.

**Variables**	**Health**		**Health**	
	**Paware**		**lninduspro**	
*pressureln*_*paware*	−0.0327***	−0.0298***		
	(0.0117)	(0.00996)		
*pressurelninduspro*			0.0142***	0.0130***
			(0.00405)	(0.00392)
ln _*grec*	−0.602**	−0.514**	−0.574**	−0.507**
	(0.220)	(0.229)	(0.212)	(0.228)
ln _*fe*	−0.915***	−0.274	−0.957***	−0.217
	(0.282)	(0.530)	(0.268)	(0.523)
ln _*edu*	1.835	0.756	1.268	0.377
	(3.476)	(3.522)	(3.470)	(3.535)
*lnlaberwag*		−0.236		−0.318
		(0.643)		(0.639)
*lnexport*		−0.705**		−0.708**
		(0.294)		(0.291)
*Policy*		0.219		0.150
		(0.160)		(0.155)
*Constant*	23.52***	27.59***	25.01***	28.15***
	(5.243)	(4.523)	(5.368)	(4.573)
*Observations*	510	510	509	509
*R*−*sq*	0.439	0.464	0.447	0.471
*Number*	30	30	30	30

Notes: ***, **, and * respectively indicates significance at the 1%, 5% and 10% level. Standard error in parentheses.

## Conclusions

This paper conducts a panel unit root test on the series of variables related to the pressure to reach economic growth targets and residents' health level, and the results show that the data are stable. Subsequently, through the nuclear density map of the economic target pressure of each province in different years, which shows that the economic growth target pressure presented a trend of first decreasing and then increasing. On this basis, this paper uses the panel fixed effects model to explore whether the impact of the pressure to reach economic growth targets on residents' health is restrained or promoted based on the panel data of 30 provinces and cities in Chinese inland provinces and regions from 2003 to 2019. The empirical results show that the pressure to reach economic growth targets has a significant inhibitory effect on residents' health, and this inhibitory effect of the northern provinces is more obvious than that of the southern provinces; In addition, the pressure to reach economic growth targets in developed areas has an insignificant effect on residents' health, while in less developed areas, the effect is stronger. Furthermore, this paper uses the logarithm of the regional PM2.5 concentration (*Pwair*) and industrial solid waste discharge (*Solid*) as the intermediary variables to verify the mechanism of the pressure to reach economic growth targets on residents' health. The results show that PM2.5 concentration and industrial solid waste emissions have a partial mediating effect. It is verified that the pressure generated by the government's economic growth target will increase industrial pollution emissions, decrease air quality, and thereby inhibit residents' health. Finally, this paper selects the public environmental awareness (*paware*) and the logarithmic form of total industrial output (*induspro*) as adjustment variables, and studies their adjustment effects in the process of the pressure to reach economic growth targets on residents' health. The results show that the increase in public awareness of environmental protection can weaken the negative impact of the pressure to reach economic growth targets on residents' health, and that the increase in industrial production will aggravate the inhibitory effect of the pressure to reach economic growth targets on residents' health. In summary, these conclusions provide a path for the government's environmental protection department and medical care department to respond to the policy planning of economic growth targets. First, the government should give more consideration to environmental factors when formulating economic growth targets, and appropriately reduce the pressure on the targets. Second, in terms of environmental governance, the government should strengthen the environmental protection policy of energy conservation and emission reduction, coordinate the relationship between production and environmental protection, and increase transfer payments for residents' health and environmental protection. Finally, the government must pay attention to the construction of the environmental protection legal system and cultivate public awareness to protect residents' health.

## Data Availability Statement

The original contributions presented in the study are included in the article/supplementary material, further inquiries can be directed to the corresponding author.

## Author Contributions

MZ: conceptualization and methodology. PW: writing and editing. MJ: writing and reviewing. X-HZ: writing-original draft. H-XW: software and data preparation. All authors contributed to the article and approved the submitted version.

## Conflict of Interest

The authors declare that the research was conducted in the absence of any commercial or financial relationships that could be construed as a potential conflict of interest.

## Publisher's Note

All claims expressed in this article are solely those of the authors and do not necessarily represent those of their affiliated organizations, or those of the publisher, the editors and the reviewers. Any product that may be evaluated in this article, or claim that may be made by its manufacturer, is not guaranteed or endorsed by the publisher.
